# The Impact of Physical Activity Intensity on the Dynamic Progression of Cardiometabolic Multimorbidity: Prospective Cohort Study Using UK Biobank Data

**DOI:** 10.2196/46991

**Published:** 2023-09-25

**Authors:** Bao-Peng Liu, Jia-Hui Zhu, Li-Peng Wan, Zhen-Yu Zhao, Xinting Wang, Cun-Xian Jia

**Affiliations:** 1 Department of Epidemiology, School of Public Health Cheeloo College of Medicine Shandong University Jinan China

**Keywords:** physical activity intensity, PA, dynamic progression, cardiometabolic multimorbidity, cohort study, CMM

## Abstract

**Background:**

Although many studies have reported on the associations between the amount of physical activity (PA) and the transitions of cardiometabolic multimorbidity (CMM), the evidence for PA intensity has not been fully evaluated.

**Objective:**

This study aimed to explore the impact of PA intensity on the dynamic progression of CMM.

**Methods:**

The prospective cohort of this study using data from the UK Biobank included 359,773 participants aged 37-73 years who were recruited from 22 centers between 2006 and 2010. The diagnoses of CMM, which included the copresence of type 2 diabetes (T2D), ischemic heart disease, and stroke, were obtained from *first occurrence fields* provided by the UK Biobank, which included data from primary care, hospital inpatient record, self-reported medical condition, and death registers. The PA intensity was assessed by the proportion of vigorous PA (VPA) to moderate to vigorous PA (MVPA). Multistate models were used to evaluate the effect of PA intensity on the dynamic progression of CMM. The first model (model A) included 5 transitions, namely free of cardiometabolic disease (CMD) to first occurrence of CMD (FCMD), free of CMD to death, FCMD to CMM, FCMD to mortality, and CMM to mortality. The other model (model B) used specific CMD, namely T2D, ischemic heart disease, and stroke, instead of FCMD and included 11 transitions in this study.

**Results:**

The mean age of the included participants (N=359,773) was 55.82 (SD 8.12) years at baseline, and 54.55% (196,271/359,773) of the participants were female. Compared with the participants with no VPA, participants with intensity levels of >0.75 to <1 for VPA to MVPA had a 13% and 27% lower risk of transition from free of CMD to FCMD (hazard ratio [HR] 0.87, 95% CI 0.83-0.91) and mortality (HR 0.73, 95% CI 0.66-0.79) in model A, respectively. The HR for the participants with no moderate PA was 0.82 (95% CI 0.73-0.92) compared with no VPA. There was a substantially protective effect of higher PA intensity on the transitions from free of CMD to T2D and from T2D to mortality, which reveals the importance of PA intensity for the transitions of T2D. More PA and greater intensity had a synergistic effect on decreasing the risk of the transitions from free of CMD to FCMD and mortality. Male participants, younger adults, adults with a higher BMI, current or previous smokers, and excessive alcohol drinkers could obtain more benefits from higher PA intensity for the lower risk of at least 1 transition from free of CMD, then to CMM, and finally to mortality.

**Conclusions:**

This study suggests that higher PA intensity is an effective measure for preventing CMM and mortality in the early period of CMM development. Relevant interventions related to higher PA intensity should be conducted.

## Introduction

### Background

Multimorbidity, defined as the coexistence of ≥2 chronic conditions from noncommunicable disease, mental disease, or infectious disease of long duration, is an unignorable public health issue that brings great physical and mental burden to families [1]. Cardiometabolic multimorbidity (CMM), namely the copresence of type 2 diabetes (T2D), ischemic heart disease (IHD), and stroke, is associated with significantly multiplicative mortality risk and lower life expectancy [2,3]. The prevalence of CMM for adults in the United States is 14.4% and is higher among the male population and older adults [4]. Previous studies characterized a troubling rise in the prevalence of CMM from 1999 to 2018 [4]. Early prevention is needed in consideration of the many adverse health outcomes related to CMM, such as cognitive decline, dementia, depression, and even a worse COVID-19 prognosis [5-8]. Previous studies have mentioned that many variables such as handgrip strength, sleep pattern, beverage consumption, shift work, and ambient air pollution are independent factors for predicting CMM [9-13]. Exploring more factors affecting the early stage of CMM, which means free of cardiometabolic disease (CMD) or first occurrence of CMD (FCMD), is necessary for preventing adverse and severe outcomes in the future.

A high level of physical activity (PA), especially for a longer duration of moderate to vigorous PA (MVPA), has been shown to be the common protective factor for CMD, including T2D [14-16], IHD [17-19], stroke [20-22], CMM [23-26], and death [27-30]. Previous studies also reported that a higher intensity of PA, namely a higher proportion of vigorous PA (VPA), could bring additional benefits for reducing the risk of mortality [31-34]. However, there is limited evidence for the associations of PA intensity with CMD and CMM. In other words, it is unclear whether a higher intensity of PA has a positive effect on decreasing the risk of mortality among the participants with CMD or CMM. Furthermore, previous studies also reported that the amount of PA, namely the duration or metabolic equivalent task (MET) for PA, could affect all the stages from healthy status to CMD, then to CMM, and finally to mortality [23,24]. To the best of our knowledge, no previous studies have evaluated the association of PA intensity with all the transitions simultaneously. Considering the studies focusing on a single transition might overlook some competing risks of mortality, future studies should investigate how PA intensity affects the dynamic progression from the free of CMD status to CMM and from other status to mortality.

### Objectives

This study aimed to explore the associations of PA intensity with the dynamic progression of CMM, namely from free of CMD to FCMD, then to CMM, and finally to mortality. In addition, the effect of PA intensity on the transitions from specific CMD, namely T2D, IHD, and stroke, to CMM and mortality was further assessed. Furthermore, the effect of modification by sociodemographic factors and the amount of PA was also assessed. The findings of this study would provide some evidence of the benefits and help relevant departments to develop some interventions related to the PA intensity for CMM and mortality.

## Methods

### Study Design and Participants

The UK Biobank (UKB) is an ongoing population-based and prospective cohort, which includes >500,000 participants aged 37 to 73 years. The details of recruitment for the UKB can be found elsewhere [35]. The main aims of the UKB are to explore genetic and nongenetic determinants of the diseases among middle-aged adults and older adults. Briefly, UKB participants were recruited from 22 centers in the United Kingdom (England, Wales, and Scotland) between 2006 and 2010 at baseline. After obtaining electronic consent for the use of deidentified data, every participant finished a self-completed touchscreen questionnaire, including socioeconomic factors and lifestyles. In addition, computer-assisted interviews, physical measurements, and sample collection were also conducted. The records from national health-related hospitals, primary care, and death registers were also matched with the included participants, which could help us comprehensively understand their health status.

In this study, we excluded participants whose records were withdrawn or could not be followed up from the cohort. In addition, the participants with missing PA data at baseline were also excluded. Finally, we excluded the participants with CMD at baseline to avoid inverse causality. Finally, 359,773 participants were included in this study (Figure S1 in Multimedia Appendix 1).

### Measures

#### Exposures

In this study, PA was assessed by using the short-form International Physical Activity Questionnaire, which has good validity and reliability [36]. The MET of moderate PA (MPA) and VPA calculated by the UKB was used in this study (data field 22038 and 22039). MVPA, which is the sum of MET associated with MPA and VPA, was used to assess the amount of PA according to the recommendations of the World Health Organization PA guidelines [37]. First, the amount of PA was categorized into <600, 600 to 1200, and >1200 MET minutes per week associated with MVPA. Second, PA intensity, which is the ratio of VPA to MVPA, was measured by using the MET of VPA to MVPA as follows: MET (VPA) / MET (MVPA). The PA intensity ranged from 0 to 1, and a higher ratio indicated higher PA intensity. Different categories (0, >0 to 0.25, >0.25 to 0.5, >0.5 to 0.75, >0.75 to <1, and 1) of PA intensity were used to assess the associations with all the transitions from the free of CMD status to FCMD, then to CMM, and finally to mortality. In addition, the ordinal scale, which coded the PA intensity from 0 to 5, was also used to measure the associations.

#### Outcomes

The incidence, progression, and prognosis of CMM, namely the occurrence of at least 2 diseases of T2D, IHD, and stroke, were the main outcomes in this study. The diagnoses and time of occurrence were obtained from *first occurrence fields* provided by UKB (data category: 2409) and were coded using the International Classification of Disease, 10th version, which included data from primary care, hospital inpatient record, self-reported medical condition, and death registers. T2D was coded by E11 and E14, as recommended in previous studies related to CMM diagnoses [9,24]. IHD was coded by I20-I25, and stroke was coded by I60-I69.

The records related to all-cause mortality were identified by linking to death registries of the National Health Service Information Centre for participants from England and Wales and the National Health Service Central Register for participants from Scotland [35]. The participants entered the cohort from the date of being recruited at the centers and exited at the date of death, occurrence of outcomes at respective progression, or censorship.

#### Covariates

The covariates in this study, which were chosen according to previous studies on CMM [9,23,24], included age, sex, ethnicity, socioeconomic status (SES), BMI, household income before tax per year, study center region, education group, smoking status, and alcohol drinking status. According to previous studies [38,39], an a priori–defined directed acyclic graph [40] was drawn to show the relationship between the covariates and outcomes (Figure S2 in Multimedia Appendix 1). Ethnicity was classified as either White or Others. The SES was measured using the Townsend area deprivation index [41]. Higher scores indicated greater socioeconomic deprivation and lower SES, and quartiles of the score for SES were included in the analyses. The data field and definition of other included covariates are shown in Table S1 in Multimedia Appendix 1. Missing values for the covariates were regarded as a classification when performing the analyses.

### Statistical Analysis

We used R (version 4.1.2; R Foundation for Statistical Computing) software to perform statistical analyses. Initially, traditional Cox proportional hazards models were used to explore the association of the amount of PA and intensity with FCMD, CMM, and all-cause mortality. No obvious violations to the proportional hazards assumption were noted for interested exposures. All the models were adjusted for age, sex, ethnicity, SES, BMI, household income before tax per year, study center region, education group, smoking status, and alcohol drinking status.

**Figure 1:**
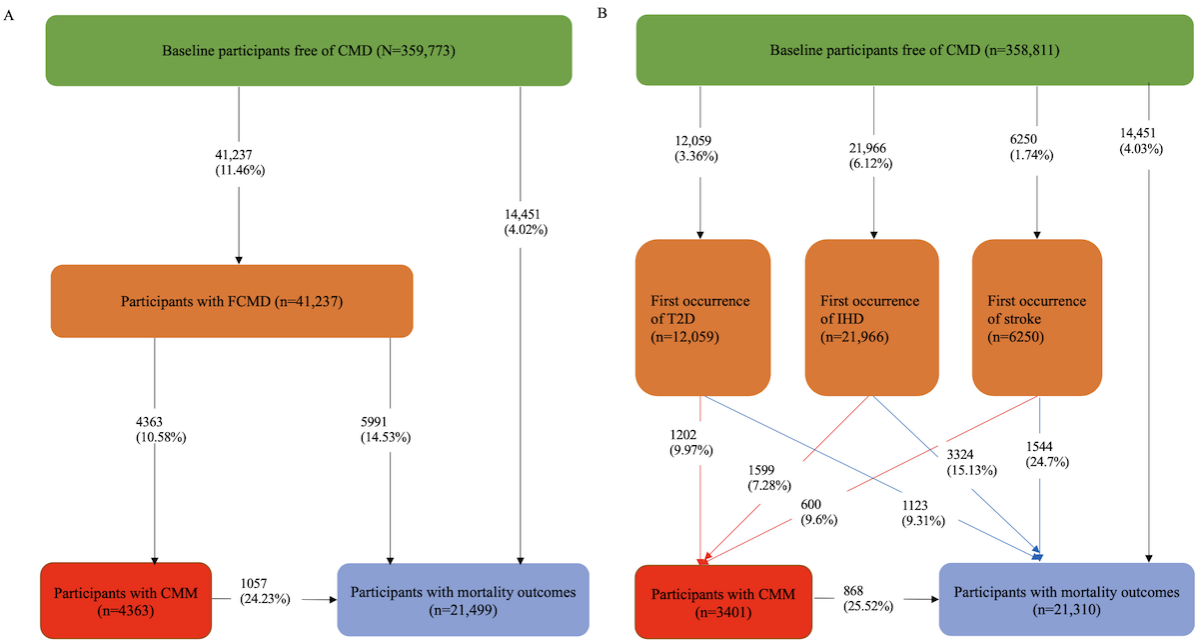
Transitions from free of cardiometabolic disease (CMD) to mortality outcomes: (A) 5-transition model and (B) 11-transition model. CMM: cardiometabolic multimorbidity; FCMD: first cardiometabolic disease; IHD: ischemic heart disease; T2D: type 2 diabetes.

Furthermore, multistate models [42,43] were used to assess the effect of PA amount and intensity on the transitions from free of CMD to FCMD, then to CMM, and finally to mortality. The multistate model, which was an extension of the traditional Cox proportional hazards model and competing risk model, was widely used to explore the effect of interested risk factors on multiple transitions related to multistate outcomes. In this study, we adopted 2 series of multistate models, as recommended by previous studies [9,23,24]. The first model (model A) included 5 transitions, namely free of CMD to FCMD, free of CMD to death, FCMD to CMM, FCMD to mortality, and CMM to mortality (Figure 1). The other model (model B) used specific CMD, namely T2D, IHD, and stroke, instead of FCMD and included 11 transitions (Figure 1). When the participants had the same date for different transitions, we adopted a theoretically time of pushing entering date forward by 0.5 day, which was based on previous studies related to a similar topic [9,24]. When performing model B, we excluded 962 participants with the same date of diagnosis for T2D, IHD, or stroke to explore disease-specific associations, as we were unsure which disease occurred first.

A series of subgroup analyses related to age (≤60 vs >60 y), sex (female vs male), BMI (underweight or normal weight vs overweight), smoking status (never vs previous or current), and alcohol drinking status (daily or almost daily vs ≤1-3 times/wk) were performed to identify the susceptible populations associated with PA intensity. *Z* test was used to compare the estimates of different subgroups, as recommended by Altman and Bland [44].

We performed several sensitivity analyses to verify the robustness of the estimates of model A. First, we used different time intervals (0.5 y, 1 y, 2 y, 3 y, and 5 y) to theoretically change the entering time to test the stability of the estimates. Second, we excluded the participants who entered different transitions on the same day. Third, we omitted the participants with FCMD for the first 2 years of follow-up to account for reverse causality. Fourth, considering the interactive effect of cancer and CMD, we reanalyzed the associations by omitting the participants with cancer at baseline. In addition, the participants with missing covariate values were excluded from the analyses. Finally, considering the correlation of air pollution with PA and CMM, we separately added particulate matter 2.5, particulate matter 10, nitric oxide, and nitrogen dioxide at baseline in the multistate models. The data of air pollution were estimated using the Land Use Regression model developed as part of the European Study of Cohorts for Air Pollution Effects and can be found in the UKB database (field id: 24003, 24004, 24005, and 24006).

### Ethical Considerations

UKB received ethics approval from the North West Multi-centre Research Ethics Committee (reference 21/NW/0157). All participants provided written informed consent before enrollment in the study, which was conducted in accordance with the principles of the Declaration of Helsinki. The data used in this study were anonymized and deidentified for privacy and confidentiality protection. More details about ethics approval can be found on the UKB website.

## Results

### Participant Characteristics at Baseline and Transitions From Baseline to Mortality

Of the included participants, 54.55% (196,271/359,773) were female and 94.91% (341,474/359,773) were White. The mean age of the participants was 55.82 (SD 8.12) years at baseline. Moreover, 64.12% (230,676/359,773) of the participants were overweight and obese, and 88.68% (319,043/359,773) of the participants were recruited from England; 56.06% (201,671/359,773) of the participants never smoked, and 70.21% (252,589/359,773) of the participants drank daily, almost daily, or more than once per week. Furthermore, 38.9% (139,963/359,773), 19.55% (70,329/359,773), and 41.55% (149,481/359,773) of the included participants had MVPA of <600, 600 to 1200, and >1200 MET minutes per week, respectively. The participants with lower PA intensity were more likely to be female; be of younger age, belong to a different ethnicity, have a lower SES, have a higher BMI, have lower income, have a lower education level, engage in more frequent drinking, and have less PA; and be current smokers. All the differences for different groups of PA intensity were statistically significant. More characteristics for included participants are shown in Table 1.

There were 2 patterns for the transitions from free of CMD to FCMD, then to CMM, and finally to mortality. The 5-transition model and 11-transition model are shown in Figure 1. For pattern A, 11.46% (41,237/359,773) of the participants developed FCMD and 4.02% (14,451/359,773) of the participants among baseline participants free of CMD died eventually. Moreover, 10.58% (4363/41,237) of the participants developed CMM, and 14.53% (5991/41,237) of the participants died after FCMD. Finally, 24.23% (1057/4363) of the participants died after a CMM diagnosis. For pattern B, 3.36% (12,059/358,811), 6.12% (21,966/358,811), and 1.74% (6250/358,811) of the participants, among the baseline participants free of CMD, first developed T2D, IHD, and stroke, respectively. Moreover, 8.44% (3401/40275) of the participants developed CMM, and 14.88% (5991/40275) of the participants died after a diagnosis of T2D, IHD, and stroke. Eventually, 25.52% (868/3401) of the participants died after having a diagnosis of CMM. More details are presented in Figure 1.

**Table 1:** Descriptive statistics by physical activity (PA) intensity (N=359,773).

Characteristics^a^			Overall		No MVPA^b^ (n=47,514)		PA intensity: the proportion of VPA^c^ to MVPA										
							0 (n=94,989)		>0 to 0.25 (n=42,934)		>0.25 to 0.5 (n=65,347)		>0.5 to 0.75 (n=75,480)		>0.75 to <1 (n=24,606)		1 (n=8903)
**Sex, n (%)**																	
	Female	196,271 (54.55)		26,608 (56)		57,040 (60.05)		24,332 (56.67)		35,951 (55.02)		36,792 (48.74)		11,154 (45.33)		4394 (49.35)	
	Male	163,502 (45.44)		20,906 (44)		37,949 (39.95)		18,602 (43.33)		29,396 (44.98)		38,688 (51.25)		13,452 (54.67)		4509 (50.64)	
Age (years), mean (SD)			55.82 (8.12)		55.60 (7.91)		57.05 (7.92)		57.54 (7.87)		56.00 (8.11)		54.45 (8.15)		53.11 (8.01)		53.47 (8.1)
**Ethnicity, n (%)**																	
	White	341,474 (94.91)		44,652 (93.98)		90,312 (95.08)		41,432 (96.5)		62,396 (95.48)		71,274 (94.43)		23,188 (94.24)		8220 (92.33)	
	Others	17,256 (4.8)		2677 (5.63)		4429 (4.66)		1386 (3.23)		2768 (4.23)		4002 (5.3)		1349 (5.48)		645 (7.24)	
	Unknown	1043 (0.29)		185 (0.39)		248 (0.26)		116 (0.27)		183 (0.28)		204 (0.27)		69 (0.28)		38 (0.04)	
**Socioeconomic status, n (%)^d^**																	
	First quartile	89,899 (24.99)		10,834 (22.8)		22,203 (23.37)		11,253 (26.21)		16,946 (25.93)		19,662 (26.05)		6722 (27.31)		2279 (25.6)	
	Second quartile	89,761 (24.95)		11,309 (23.8)		23,192 (24.41)		11,122 (25.9)		16,653 (25.48)		18,996 (25.17)		6341 (25.77)		2148 (24.13)	
	Third quartile	89,832 (24.97)		11,689 (24.6)		23,831 (25.09)		10,849 (25.27)		16,404 (25.1)		18,852 (24.98)		6009 (24.42)		2198 (24.69)	
	Fourth quartile	89,827 (24.97)		13,613 (28.65)		25,640 (26.99)		9666 (22.51)		15,264 (23.36)		17,874 (23.68)		5501 (22.36)		2269 (25.48)	
	Unknown	454 (0.13)		69 (0.14)		123 (0.13)		44 (0.1)		80 (0.12)		96 (0.13)		33 (0.13)		9 (0.1)	
**BMI (kg/m^2^), n (%)**																	
	Underweight (<18.5)	1939 (0.54)		296 (0.62)		638 (0.67)		244 (0.57)		325 (0.5)		313 (0.41)		95 (0.39)		28 (0.31)	
	Normal weight (18.5-24.9)	125,609 (34.91)		12,981 (27.32)		30,952 (32.58)		16,229 (37.8)		24,794 (37.94)		28,015 (37.11)		9536 (38.75)		3102 (34.84)	
	Overweight (25.0-29.9)	154,404 (42.92)		19,466 (40.97)		40,039 (42.15)		18,632 (43.4)		28,244 (43.22)		33,311 (44.13)		10,843 (44.07)		3869 (43.46)	
	Obese (≥30)	76,272 (21.2)		14,359 (30.22)		22,929 (24.14)		7691 (17.91)		11,774 (18.02)		13,574 (17.98)		4076 (16.56)		1869 (20.99)	
	Unknown	1549 (0.43)		412 (0.87)		431 (0.45)		138 (0.32)		210 (0.32)		267 (0.35)		56 (0.23)		35 (0.39)	
**Household income before tax/y (£^e^), n (%)**																	
	<18,000	62,366 (17.33)		9501 (20.15)		19,972 (21.02)		8107 (18.88)		10,569 (16.17)		10,553 (13.98)		2556 (10.39)		1108 (12.44)	
	18,000-30,999	78,380 (21.78)		9601 (20.21)		21,950 (23.11)		11,395 (26.54)		14,942 (22.86)		14,902 (19.74)		4174 (16.96)		1416 (15.9)	
	31,000-51,999	86,831 (24.13)		11,069 (23.3)		21,890 (23.04)		10,544 (24.56)		16,194 (24.78)		18,796 (24.9)		6184 (25.13)		2154 (24.19)	
	52,000-100,000	71,816 (19.96)		9043 (19.03)		15,859 (16.7)		6560 (15.28)		12,909 (19.75)		17,951 (23.78)		7112 (28.9)		2382 (26.75)	
	>100,000	19,873 (5.52)		2129 (4.48)		3726 (3.92)		1551 (3.61)		3532 (5.4)		5634 (7.46)		2557 (10.39)		744 (8.36)	
	Unknown	40,507 (11.26)		6171 (12.99)		11,592 (12.2)		4777 (11.13)		7201 (11.02)		7644 (10.13)		2023 (8.22)		1099 (12.34)	
**Study center region, n (%)**																	
	England	319,043 (88.68)		41,593 (87.54)		83,956 (88.38)		38,619 (89.95)		58,252 (89.15)		66,963 (88.72)		21,773 (88.49)		7887 (88.59)	
	Wales	14,829 (4.12)		2309 (4.86)		3965 (4.17)		1639 (3.82)		2496 (3.82)		3057 (4.05)		996 (4.05)		367 (4.12)	
	Scotland	25,901 (7.2)		3612 (7.6)		7068 (7.44)		2676 (6.23)		4599 (7.04)		5460 (7.23)		1837 (7.46)		649 (7.29)	
**Education group, n (%)**																	
	College or university degree	129,911 (36.11)		14,438 (30.39)		30,719 (32.34)		14,510 (33.8)		24,849 (38.03)		30,087 (39.86)		11,581 (47.06)		3727 (41.86)	
	Any school degree (A-level^f^, AS-level^g^, O-level^h^, GCSE^i^, and CSE^j^)	138,483 (38.49)		19,240 (40.49)		37,065 (39.02)		17,251 (40.18)		24,817 (37.98)		28,106 (37.24)		8707 (35.38)		3297 (37.03)	
	Vocational qualification (NVQ^k^, HND^l^, or HNC^m^) or other professional qualifications	40,545 (11.27)		5054 (10.64)		10,947 (11.52)		5524 (12.87)		7747 (11.85)		8154 (10.8)		2329 (9.46)		790 (8.87)	
	None of the above	48,412 (13.46)		8351 (17.57)		15,565 (16.39)		5414 (12.61)		7548 (11.55)		8644 (11.45)		1878 (7.63)		1012 (11.37)	
	Unknown	2422 (0.67)		431 (0.91)		693 (0.73)		235 (0.55)		386 (0.59)		489 (0.65)		111 (0.45)		77 (0.86)	
**Smoking status, n (%)**																	
	Never	201,671 (56.06)		25,457 (53.58)		51,901 (54.64)		23,844 (55.54)		37,116 (56.8)		43,488 (57.61)		14,705 (59.76)		5160 (57.96)	
	Previous	121,014 (33.64)		15,493 (32.61)		31,669 (33.34)		15,045 (35.04)		22,407 (34.29)		25,374 (33.61)		8061 (32.76)		2965 (33.3)	
	Current	36,181 (10.06)		6385 (13.44)		11,144 (11.73)		3951 (9.2)		5706 (8.73)		6454 (8.55)		1787 (7.26)		754 (8.47)	
	Unknown	907 (0.25)		179 (0.38)		275 (0.29)		94 (0.22)		118 (0.18)		164 (0.22)		53 (0.21)		24 (0.27)	
**Alcohol drinking status, n (%)**																	
	Daily or almost daily	77,268 (21.48)		9664 (20.34)		20,644 (21.73)		10,029 (23.36)		14,441 (22.1)		15,753 (20.87)		5028 (20.43)		1709 (19.19)	
	Above 1 time/wk	181,021 (50.31)		21,422 (45.08)		44,118 (46.44)		21,470 (50.01)		34,130 (52.23)		41,242 (54.64)		13,925 (56.59)		4714 (52.95)	
	1-3 times/mo	39,390 (10.95)		5740 (12.08)		10,895 (11.47)		4604 (10.72)		6943 (10.62)		7664 (10.15)		2563 (10.42)		981 (11.02)	
	Special occasions only	37,073 (10.3)		6042 (12.72)		11,479 (12.08)		4323 (10.07)		5969 (9.13)		6511 (8.63)		1850 (7.52)		899 (10.1)	
	Never	24,830 (6.9)		4601 (9.68)		7791 (8.2)		2496 (5.81)		3840 (5.88)		4277 (5.67)		1231 (5)		594 (6.67)	
	Unknown	191 (0.05)		45 (0.09)		62 (0.06)		12 (0.03)		24 (0.04)		33 (0.04)		9 (0.04)		6 (0.07)	
**PA amount (MET^n^ min/wk), n (%)**																	
	<600	139,963 (38.9)		47,514 (100)		58,656 (61.75)		3780 (8.8)		12,729 (19.48)		10,695 (14.17)		2726 (11.08)		3863 (43.39)	
	600-1200	70,329 (19.55)		0 (0)		17,960 (18.91)		9121 (21.24)		16,019 (24.51)		17,574 (23.25)		7118 (28.93)		2537 (28.5)	
	>1200	149,481 (41.55)		0 (0)		18,373 (19.34)		30,033 (69.95)		36,599 (56.01)		47,211 (62.5)		14,762 (60)		2503 (28.11)	

^a^Unknown included prefer not to answer, do not know, and missing values in the UK Biobank database.

^b^MVPA: moderate to vigorous PA.

^c^VPA: vigorous PA.

^d^Socioeconomic status was measured by using the Townsend area deprivation index.

^e^A currency exchange rate of £1=US $1.26 is applicable.

^f^A-level: Advanced level.

^g^AS-level: Advanced Subsidiary level.

^h^O-level: Ordinary level.

^i^GCSE: General Certificate of Secondary Education.

^j^CSE: General Certificate of Education.

^k^NVQ: National Vocational Qualification.

^l^HND: Higher National Diploma.

^m^HNC: Higher National Certificate.

^n^MET: metabolic equivalent task.

### Traditional Cox Proportional Hazards Model

The estimates of the associations of PA amount and intensity with FCMD, CMM, and mortality are presented in Table S2 in Multimedia Appendix 1. More MVPA with 600-1200 and >1200 MET minutes per week lowered the risk of FCMD, CMM, and mortality than MVPA with <600 MET minutes per week. A decreased risk of FCMD, CMM, and mortality was also found in the participants with more VPA to MVPA (>0 to <1) than in the participants with no VPA. However, the associations of no MPA, namely total VPA with FCMD (*P*=.073), CMM (*P*=.294), and mortality (*P*=.123), were not statistically significant compared with no VPA. Similar results were found when the ordinal scale of PA intensity was used in the models.

### Multistate Models

Multistate models for the associations of PA amount (Table S3 in Multimedia Appendix 1) and intensity (Table 2) with all the transitions were performed. First, meeting the recommendations of the World Health Organization, namely no less than 600 MET minutes per week for MVPA were found to be negatively associated with transitions from free of CMD to FCMD and mortality. In addition, >1200 MET minutes per week for MVPA was significantly associated with a decreased risk of transition from FCMD to CMM. When focusing on specific FCMD, more PA was found to be associated with a decreased risk of transitions from free of CMD to T2D, IHD, stroke, and mortality as well as transitions from stroke to CMM and from T2D to mortality.

A greater proportion of VPA to MPA, namely >0.5 for the proportion of VPA to MVPA, was associated with a lower risk of different transitions. Our analyses showed that compared with the participants with no VPA, participants with >0.75 to <1 of VPA to MVPA had a 13% and 27% lower risk of transition from free of CMD to FCMD (hazard ratio [HR] 0.87, 95% CI 0.83-0.91) and mortality (HR 0.73, 95% CI 0.66-0.79), respectively. Furthermore, the HR for the participants with no MPA was 0.82 (95% CI 0.73-0.92) compared with those with no VPA. The participants with >0.75 to <1 VPA to MVPA had the lowest risk of transitions from free of CMD to FCMD and mortality. The decreased risk of transitions from free of CMD to FCMD and mortality was also related to higher PA intensity in consideration of the ordinal scale. However, we did not find significant associations of PA intensity with other transitions in model A.

Model B showed different associations of PA intensity with the transitions related to specific FCMD. Specifically, a greater proportion of VPA to MPA was associated with a lower risk of transitions from free of CMD to T2D and stroke. In addition, a decreased risk of transitions from stroke to CMM and from T2D to mortality was found to be associated with >0.5 to 0.75 of VPA to MVPA. It was noted that lower PA intensity, that is, >0 to 0.5 of VPA to MVPA, was also found to be associated with a lower risk of transition from T2D to mortality. The ordinal scale of higher PA intensity was significantly associated with a lower risk of transition from free of CMD to T2D, stroke, and mortality.

**Table 2:** Associations of physical activity (PA) intensity with transitions from free of cardiometabolic disease (CMD) to first occurrence of CMD (FCMD), cardiometabolic multimorbidity (CMM), and mortality.

Transitions			PA intensity: the proportion of VPA^a^ to MVPA^b^, HR^c^ (95% CI)												
			0		>0 to 0.25		>0.25 to 0.5		>0.5 to 0.75		>0.75 to <1		1		Ordinal scale
**Model A^d^**															
	From free of CMD to FCMD	Reference		0.90 (0.87-0.93)^e^		0.90 (0.87-0.92)^e^		0.89 (0.87-0.92)^e^		0.87 (0.83-0.91)^e^		0.97 (0.90-1.03)		0.97 (0.97-0.98)^e^	
	From free of CMD to mortality	Reference		0.90 (0.85-0.95)^e^		0.85 (0.81-0.89)^e^		0.80 (0.76-0.84)^e^		0.73 (0.67-0.79)^e^		0.82 (0.73-0.93)^f^		0.94 (0.92-0.95)^e^	
	From FCMD to CMM	Reference		0.91 (0.82-1.01)		0.98 (0.89-1.08)		0.91 (0.83-1.00)		1.04 (0.90-1.21)		1.05 (0.85-1.30)		1.00 (0.97-1.02)	
	From FCMD to mortality	Reference		0.93 (0.85-1.02)		0.92 (0.85-1.00)		0.93 (0.86-1.01)		0.94 (0.83-1.08)		0.98 (0.82-1.18)		0.99 (0.97-1.01)	
	From CMM to mortality	Reference		1.07 (0.86-1.32)		0.97 (0.80-1.18)		1.04 (0.85-1.26)		0.93 (0.66-1.32)		1.16 (0.76-1.77)		1.01 (0.96-1.06)	
**Model B** ^d^															
	From free of CMD to T2D^g^	Reference		0.74 (0.69-0.79)^e^		0.77 (0.72-0.81)^e^		0.77 (0.73-0.82)^e^		0.73 (0.66-0.79)^e^		0.99 (0.88-1.12)		0.94 (0.93-0.95)^e^	
	From free of CMD to IHD^h^	Reference		0.96 (0.91-1.00)		0.95 (0.91-0.99)^i^		0.97 (0.93-1.01)		0.95 (0.89-1.01)		1.02 (0.93-1.11)		1.00 (0.99-1.01)	
	From free of CMD to stroke	Reference		1.06 (0.97-1.15)		0.97 (0.90-1.05)		0.91 (0.85-0.99)^i^		0.91 (0.81-1.03)		0.75 (0.62-0.92)^f^		0.97 (0.95-0.99)^f^	
	From free of CMD to mortality	Reference		0.90 (0.85-0.95)^e^		0.85 (0.81-0.89)^e^		0.80 (0.76-0.84)^e^		0.73 (0.67-0.79)^e^		0.82 (0.73-0.93)^f^		0.94 (0.92-0.95)^e^	
	From T2D to CMM	Reference		0.87 (0.70-1.08)		1.01 (0.84-1.21)		0.98 (0.82-1.18)		0.91 (0.66-1.25)		1.12 (0.76-1.64)		1.00 (0.96-1.05)	
	From IHD to CMM	Reference		0.96 (0.78-1.18)		0.84 (0.69-1.02)		0.95 (0.80-1.14)		0.74 (0.52-1.05)		1.04 (0.70-1.55)		0.97 (0.93-1.02)	
	From stroke to CMM	Reference		0.94 (0.79-1.10)		0.89 (0.76-1.04)		0.84 (0.72-0.98)^i^		0.96 (0.75-1.22)		1.09 (0.78-1.53)		0.98 (0.94-1.01)	
	From T2D to mortality	Reference		0.80 (0.71-0.90)^e^		0.86 (0.77-0.95)^f^		0.87 (0.79-0.97)^f^		1.03 (0.87-1.21)		1.08 (0.86-1.36)		0.99 (0.96-1.01)	
	From IHD to mortality	Reference		0.82 (0.62-1.08)		0.98 (0.77-1.25)		0.90 (0.70-1.16)		1.30 (0.91-1.86)		0.84 (0.41-1.70)		1.01 (0.95-1.08)	
	From stroke to mortality	Reference		1.09 (0.93-1.28)		1.05 (0.90-1.22)		1.01 (0.86-1.17)		0.78 (0.59-1.04)		0.77 (0.48-1.23)		0.98 (0.94-1.02)	
	From CMM to mortality	Reference		0.95 (0.75-1.21)		0.92 (0.75-1.15)		1.00 (0.81-1.24)		0.92 (0.63-1.34)		1.08 (0.66-1.76)		1.00 (0.95-1.05)	

^a^VPA: vigorous PA.

^b^MVPA: moderate to vigorous PA.

^c^HR: hazard ratio.

^d^The models were adjusted for age, sex, ethnicity, socioeconomic status, BMI, household income, study center region, education group, smoking status, alcohol drinking status, and PA amount. Model A included 5 transitions from free of CMD to FCMD, then to CMM, and finally to mortality (Figure 1A), and model B included 11 transitions by including specific FCMD (Figure 1B).

^e^*P*<.001.

^f^*P*<.01.

^g^T2D: type 2 diabetes.

^h^IHD: ischemic heart disease.

^i^*P*<.05.

### Subgroup Analyses

Several important findings were involved in the subgroup analyses of PA amount, and details are presented in Table 3. First, lower PA (<600 MET min/wk) accompanied by lower PA intensity, that is, >0 to 0.25 of VPA to MVPA, was not significantly associated with a decreased risk of any transition compared with no VPA. Second, the participants with a greater proportion of VPA to MPA were highly associated with a reduced risk of transitions from free of CMD to FCMD and mortality regardless of the amount of PA. Third, higher PA intensity was associated with a decreased risk of transitions from free of CMD to T2D and stroke for the participants with the recommended amount of PA (no less than 600 MET min/wk) and IHD for the participants under the threshold of the recommended amount of PA (<600 MET min/wk). These findings were similar when using the ordinal scale of PA intensity.

A significant effect of modification related to age, sex, PA amount, BMI, smoking, and alcohol drinking status was found among at least 1 transition (Table 4; Tables S4 and S5 in Multimedia Appendix 1). The protective effect of higher PA intensity on the transition from free of CMD to FCMD was larger among younger participants, participants with overweight, and previous or current smokers. Specifically, the decreased risk of the transitions from free of CMD to IHD and stroke associated with higher PA intensity was only found in previous or current smokers. Male participants, current or previous smokers, excessive alcohol drinkers, and participants with more PA could have more benefits of higher PA intensity for reducing mortality from free of CMD. In addition, the protective effect of higher PA intensity on the transitions from FCMD to mortality was more pronounced among the current or previous smokers. Younger populations aged ≤60 years could benefit from higher PA intensity when having CMM.

**Table 3:** Associations of physical activity (PA) intensity with transitions from free of cardiometabolic disease (CMD) to first occurrence of CMD (FCMD), cardiometabolic multimorbidity (CMM), and mortality by PA amount.

Transitions				PA intensity: the proportion of VPA^a^ to MVPA^b^, HR^c^ (95% CI)												
				0		>0 to 0.25		>0.25 to 0.5		>0.5 to 0.75		>0.75 to <1		1		Ordinal scale
**<600 MET^d^ min/wk**																
	**Model A^e^**															
		From free of CMD to FCMD	Reference		0.96 (0.87-1.06)		0.88 (0.83-0.94)^f^		0.89 (0.83-0.95)^f^		0.89 (0.78-1.02)		1.04 (0.94-1.14)		0.98 (0.96-0.99)^g^	
		From free of CMD to mortality	Reference		0.91 (0.76-1.07)		0.85 (0.76-0.94)^g^		0.76 (0.67-0.85)^f^		0.69 (0.53-0.88)^g^		0.87 (0.73-1.04)		0.94 (0.91-0.96)^f^	
		From FCMD to CMM	Reference		1.28 (0.97-1.69)		0.82 (0.66-1.01)		0.75 (0.59-0.96)^h^		1.16 (0.75-1.80)		0.93 (0.68-1.27)		0.96 (0.92-1.00)	
		From FCMD to mortality	Reference		1.07 (0.83-1.37)		0.97 (0.82-1.14)		0.90 (0.74-1.08)		1.08 (0.74-1.57)		0.91 (0.70-1.19)		0.98 (0.95-1.02)	
		From CMM to mortality	Reference		0.77 (0.40-1.51)		0.97 (0.61-1.54)		1.15 (0.70-1.89)		0.82 (0.26-2.56)		1.33 (0.74-2.39)		1.03 (0.94-1.13)	
	**Model B^e^**															
		From free of CMD to T2D^i^	Reference		0.94 (0.79-1.12)		0.81 (0.73-0.91)^f^		0.90 (0.81-1.02)		0.87 (0.69-1.10)		1.14 (0.97-1.34)		0.98 (0.96-1.01)	
		From free of CMD to IHD^j^	Reference		0.93 (0.81-1.07)		0.90 (0.83-0.98)^h^		0.87 (0.80-0.96)^g^		0.90 (0.75-1.08)		1.03 (0.90-1.18)		0.98 (0.96-1.00)^h^	
		From free of CMD to stroke	Reference		1.09 (0.85-1.39)		0.99 (0.85-1.16)		0.94 (0.79-1.12)		0.90 (0.64-1.28)		0.85 (0.64-1.12)		0.98 (0.94-1.01)	
		From free of CMD to mortality	Reference		0.91 (0.76-1.07)		0.85 (0.76-0.94)^g^		0.76 (0.67-0.85)^f^		0.69 (0.54-0.88)^g^		0.87 (0.73-1.04)		0.94 (0.91-0.96)^f^	
		From T2D to CMM	Reference		1.07 (0.62-1.83)		0.89 (0.61-1.31)		0.75 (0.49-1.17)		1.33 (0.63-2.83)		1.01 (0.60-1.70)		0.98 (0.90-1.06)	
		From IHD to CMM	Reference		1.33 (0.82-2.14)		0.81 (0.54-1.20)		0.82 (0.54-1.24)		0.57 (0.18-1.80)		0.88 (0.51-1.55)		0.95 (0.87-1.03)	
		From stroke to CMM	Reference		0.94 (0.54-1.63)		0.76 (0.53-1.08)		0.72 (0.48-1.08)		0.99 (0.44-2.22)		0.80 (0.46-1.39)		0.93 (0.86-1.00)	
		From T2D to mortality	Reference		0.85 (0.57-1.25)		0.81 (0.64-1.02)		0.81 (0.62-1.05)		1.41 (0.90-2.19)		1.00 (0.71-1.40)		0.98 (0.93-1.03)	
		From IHD to mortality	Reference		2.60 (1.46-4.64)^g^		0.61 (0.32-1.16)		0.62 (0.29-1.34)		0.64 (0.16-2.61)		1.10 (0.45-2.71)		0.93 (0.81-1.06)	
		From stroke to mortality	Reference		1.43 (0.90-2.29)		1.41 (1.06-1.88)^h^		1.24 (0.87-1.75)		0.70 (0.29-1.71)		0.93 (0.49-1.75)		1.04 (0.97-1.12)	
		From CMM to mortality	Reference		0.81 (0.38-1.73)		1.15 (0.71-1.88)		1.30 (0.76-2.21)		1.18 (0.38-3.72)		1.53 (0.78-3.00)		1.08 (0.98-1.20)	
**600-1200 MET min/wk**																
	**Model A^e^**															
		From free of CMD to FCMD	Reference		0.88 (0.81-0.95)^f^		0.88 (0.83-0.94)^f^		0.91 (0.85-0.97) ^g^		0.95 (0.87-1.04)		0.96 (0.84-1.09)		0.98 (0.97-1.00)^h^	
		From free of CMD to mortality	Reference		0.95 (0.84-1.07)		0.89 (0.80-0.99)^h^		0.85 (0.77-0.95)^g^		0.80 (0.68-0.94)^g^		0.93 (0.75-1.17)		0.96 (0.93-0.98)^g^	
		From FCMD to CMM	Reference		0.86 (0.67-1.09)		0.98 (0.81-1.20)		0.99 (0.81-1.20)		0.86 (0.63-1.17)		1.39 (0.95-2.03)		1.01 (0.96-1.06)	
		From FCMD to mortality	Reference		0.89 (0.72-1.09)		0.92 (0.77-1.09)		0.92 (0.77-1.09)		0.97 (0.76-1.25)		0.98 (0.68-1.42)		0.99 (0.95-1.03)	
		From CMM to mortality	Reference		0.84 (0.49-1.44)		0.97 (0.64-1.46)		1.02 (0.68-1.54)		1.20 (0.64-2.27)		0.97 (0.45-2.12)		1.02 (0.92-1.12)	
	**Model B^e^**															
		From free of CMD to T2D	Reference		0.77 (0.66-0.89)^f^		0.72 (0.63-0.81)^f^		0.84 (0.75-0.95)^g^		0.87 (0.74-1.04)		0.92 (0.72-1.18)		0.96 (0.93-0.99)^h^	
		From free of CMD to IHD	Reference		0.90 (0.81-1.00)^h^		0.94 (0.86-1.02)		0.97 (0.89-1.06)		1.03 (0.91-1.16)		1.05 (0.87-1.25)		1.00 (0.98-1.03)	
		From free of CMD to stroke	Reference		1.04 (0.87-1.25)		0.97 (0.83-1.14)		0.84 (0.71-1.00)^h^		0.89 (0.70-1.13)		0.71 (0.48-1.06)		0.95 (0.91-0.99)^h^	
		From free of CMD to mortality	Reference		0.95 (0.84-1.07)		0.89 (0.80-0.99)^h^		0.86 (0.77-0.95)^g^		0.80 (0.68-0.94)^g^		0.94 (0.75-1.18)		0.96 (0.93-0.98)^g^	
		From T2D to CMM	Reference		1.09 (0.70-1.72)		0.90 (0.59-1.36)		1.05 (0.71-1.53)		0.56 (0.26-1.21)		1.43 (0.66-3.10)		0.98 (0.89-1.08)	
		From IHD to CMM	Reference		0.95 (0.57-1.59)		1.08 (0.71-1.65)		1.17 (0.78-1.75)		1.02 (0.54-1.93)		1.63 (0.75-3.55)		1.05 (0.96-1.16)	
		From stroke to CMM	Reference		0.77 (0.52-1.14)		0.70 (0.50-0.99)^h^		0.92 (0.67-1.26)		0.83 (0.52-1.34)		1.50 (0.86-2.62)		1.00 (0.92-1.08)	
		From T2D to mortality	Reference		0.75 (0.57-1.00)		0.81 (0.64-1.02)		0.83 (0.65-1.04)		1.02 (0.75-1.40)		1.19 (0.77-1.85)		0.99 (0.93-1.05)	
		From IHD to mortality	Reference		0.45 (0.20-1.01)		0.96 (0.57-1.63)		0.86 (0.48-1.52)		1.09 (0.51-2.34)		0.76 (0.18-3.14)		1.00 (0.87-1.15)	
		From stroke to mortality	Reference		1.01 (0.70-1.44)		0.94 (0.69-1.30)		0.98 (0.70-1.36)		0.74 (0.42-1.29)		0.14 (0.02-1.03)		0.94 (0.86-1.02)	
		From CMM to mortality	Reference		0.62 (0.32-1.21)		0.72 (0.43-1.22)		1.02 (0.65-1.61)		1.29 (0.64-2.60)		0.90 (0.36-2.25)		1.01 (0.90-1.13)	
**>1200 MET min/wk**																
	**Model A^e^**															
		From free of CMD to FCMD	Reference		0.91 (0.87-0.96)^f^		0.92 (0.88-0.97)^g^		0.91 (0.86-0.95)^f^		0.85 (0.79-0.91)^f^		0.86 (0.75-0.99)^h^		0.97 (0.96-0.98)^f^	
		From free of CMD to mortality	Reference		0.83 (0.76-0.90)^f^		0.79 (0.73-0.86)^f^		0.75 (0.69-0.81)^f^		0.67 (0.59-0.75)^f^		0.62 (0.48-0.80)^f^		0.92 (0.90-0.93)^f^	
		From FCMD to CMM	Reference		0.88 (0.76-1.03)		1.03 (0.89-1.20)		0.93 (0.80-1.07)		1.11 (0.90-1.36)		0.95 (0.60-1.52)		1.01 (0.97-1.05)	
		From FCMD to mortality	Reference		0.93 (0.82-1.06)		0.92 (0.81-1.04)		0.95 (0.84-1.07)		0.91 (0.76-1.09)		1.14 (0.80-1.62)		0.99 (0.96-1.02)	
		From CMM to mortality	Reference		1.07 (0.80-1.44)		0.89 (0.66-1.19)		0.93 (0.70-1.25)		0.77 (0.48-1.23)		1.02 (0.37-2.78)		0.95 (0.88-1.03)	
	**Model B^e^**															
		From free of CMD to T2D	Reference		0.79 (0.71-0.87)^f^		0.86 (0.78-0.94)^g^		0.80 (0.73-0.88)^f^		0.71 (0.62-0.82)^f^		0.84 (0.64-1.09)		0.95 (0.93-0.97)^f^	
		From free of CMD to IHD	Reference		0.98 (0.91-1.05)		0.98 (0.92-1.05)		0.99 (0.93-1.06)		0.93 (0.85-1.02)		0.97 (0.81-1.16)		0.99 (0.98-1.01)	
		From free of CMD to stroke	Reference		0.93 (0.82-1.04)		0.86 (0.76-0.96)^h^		0.82 (0.73-0.93)^g^		0.82 (0.69-0.96)^h^		0.62 (0.42-0.91)^h^		0.94 (0.91-0.97)^f^	
		From free of CMD to mortality	Reference		0.83 (0.76-0.90)^f^		0.79 (0.73-0.86)^f^		0.75 (0.69-0.81)^f^		0.67 (0.59-0.75)^f^		0.62 (0.48-0.80)^f^		0.92 (0.90-0.93)^f^	
		From T2D to CMM	Reference		0.75 (0.54-1.03)		1.04 (0.78-1.38)		0.97 (0.73-1.29)		0.94 (0.61-1.44)		1.13 (0.49-2.60)		1.02 (0.95-1.10)	
		From IHD to CMM	Reference		0.83 (0.62-1.12)		0.73 (0.54-0.98)^h^		0.86 (0.65-1.13)		0.62 (0.38-1.01)		1.05 (0.46-2.39)		0.94 (0.87-1.02)	
		From stroke to CMM	Reference		1.04 (0.81-1.33)		1.05 (0.83-1.34)		0.89 (0.70-1.13)		1.05 (0.75-1.48)		1.18 (0.60-2.34)		0.98 (0.92-1.05)	
		From T2D to mortality	Reference		0.86 (0.72-1.02)		0.94 (0.79-1.11)		0.96 (0.81-1.12)		1.03 (0.82-1.30)		1.15 (0.73-1.82)		1.02 (0.97-1.06)	
		From IHD to mortality	Reference		0.74 (0.49-1.10)		1.06 (0.74-1.53)		0.95 (0.66-1.36)		1.47 (0.91-2.37)		0.44 (0.06-3.18)		1.06 (0.96-1.17)	
		From stroke to mortality	Reference		1.11 (0.87-1.41)		0.99 (0.78-1.27)		1.00 (0.79-1.26)		0.82 (0.56-1.21)		1.27 (0.59-2.73)		0.98 (0.92-1.04)	
		From CMM to mortality	Reference		0.87 (0.62-1.20)		0.78 (0.57-1.07)		0.79 (0.58-1.09)		0.64 (0.38-1.07)		0.66 (0.21-2.10)		0.92 (0.84-1.00)	

^a^VPA: vigorous PA.

^b^MVPA: moderate to vigorous PA.

^c^HR: hazard ratio.

^d^MET: metabolic equivalent task.

^e^The models were adjusted for age, sex, ethnicity, socioeconomic status, BMI, household income, study center region, education group, smoking status, alcohol drinking status, and PA amount. Model A included 5 transitions from free of CMD to FCMD, then to CMM, and finally to mortality (Figure 1A), and model B included 11 transitions by including specific FCMD (Figure 1B).

^f^*P*<.001.

^g^*P*<.01.

^h^*P*<.05.

^i^T2D: type 2 diabetes.

^j^IHD: ischemic heart disease.

**Table 4:** Subgroup analyses for the associations of physical activity (PA) intensity with transitions from free of cardiometabolic disease (CMD) to first occurrence of CMD (FCMD), cardiometabolic multimorbidity (CMM), and mortality.

Factors for subgroup analyses			Model A^a^, HR^b^ (95% CI)								
			From free of CMD to FCMD		From free of CMD to mortality		From FCMD to CMM		From FCMD to mortality		From CMM to mortality
**Age (years)**											
	≤60	0.95 (0.94-0.96)^c^		0.92 (0.90-0.94)^c^		0.97 (0.93-1.00)		0.96 (0.93-1.00)^d^		0.89 (0.81-0.98)^d^	
	>60	0.97 (0.96-0.98)^c^		0.94 (0.93-0.96)^c^		0.98 (0.95-1.01)		0.98 (0.95-1.00)		1.05 (0.99-1.11)	
**Sex**											
	Male	0.97 (0.96-0.98)^c^		0.92 (0.91-0.94)^c^		1.01 (0.98-1.04)		0.98 (0.96-1.01)		1.03 (0.98-1.10)	
	Female	0.97 (0.95-0.98)^c^		0.96 (0.94-0.98)^c^		0.99 (0.95-1.03)		1.01 (0.98-1.04)		0.97 (0.89-1.06)	
**BMI (kg/m^2^)**											
	<25	0.99 (0.97-1.00)		0.93 (0.91-0.95)^c^		0.98 (0.93-1.04)		0.97 (0.94-1.01)		0.93 (0.83-1.04)	
	≥25	0.97 (0.96-0.98)^c^		0.94 (0.92-0.95)^c^		0.99 (0.97-1.02)		0.99 (0.96-1.01)		1.02 (0.97-1.08)	
**Smoking status**											
	Never	0.99 (0.97-1.00)^e^		0.95 (0.93-0.96)^c^		1.00 (0.97-1.04)		1.01 (0.98-1.04)		0.99 (0.92-1.07)	
	Previous or current	0.96 (0.95-0.97)^c^		0.92 (0.91-0.94)^c^		0.98 (0.95-1.02)		0.96 (0.94-0.99)^e^		1.02 (0.96-1.08)	
**Alcohol drinking status**											
	≤1-3 times/wk	0.97 (0.96-0.98)^c^		0.94 (0.93-0.96)^c^		0.99 (0.96-1.02)		0.99 (0.97-1.01)		0.98 (0.93-1.04)	
	Daily or almost daily	0.98 (0.96-0.99)^e^		0.91 (0.89-0.93)^c^		1.01 (0.96-1.07)		0.97 (0.93-1.01)		1.08 (0.97-1.19)	

^a^The models were adjusted for age, sex, ethnicity, socioeconomic status, BMI, household income, study center region, education group, smoking status, alcohol drinking status, and PA amount. Age, sex, BMI, smoking status, and alcohol drinking status were not included in the models for the subgroup analyses. Model A included 5 transitions from free of CMD to FCMD, then to CMM, and finally to mortality (Figure 1A). PA intensity refers to the proportion of vigorous PA to moderate to vigorous PA, and an ordinal scale of PA intensity was used in the subgroup analyses.

^b^HR: hazard ratio.

^c^*P*<.001.

^d^*P*<.05.

^e^*P*<.01.

### Sensitivity Analysis

We did not find substantial changes for the associations of PA intensity with the transitions from free of CMD to FCMD, then to CMM, and finally to mortality by performing additional analyses (Table S6 in Multimedia Appendix 1) using different time intervals (0.5 y, 1 y, 2 y, 3 y, and 5 y) for the participants entering different transitions on the same day, excluding the participants who entered different transitions on the same day, excluding FCMD in the first 2-year follow-up, excluding the participants with cancer, and excluding the participants with missing values at baseline for the covariates, which revealed robust estimates in this study.

## Discussion

### Principal Findings

This is the first prospective cohort study using large-scale population–based data to explore the associations of PA intensity with different transitions from free of CMD, then to CMM, and finally to mortality. This study reported the effect of the proportion of VPA to MVPA on the incidence, progression, and prognosis of CMM using multistate models, and the principal findings are discussed next. Compared with the participants with no VPA, the participants with >0.75 to <1 of VPA to MVPA had the lowest risk of transitions from free of CMD to FCMD and mortality regardless of model A or B. The protective effect of higher PA intensity on the transitions from free of CMD to T2D (*P*<.001) and from T2D to mortality (*P*<.001) was statistically significant, which revealed the importance of PA intensity for the transitions of T2D. More PA and greater intensity had a synergistic effect on decreasing the risk of transitions from free of CMD to FCMD and mortality. Male participants, younger adults, participants with a higher BMI, current or previous smokers, and excessive alcohol drinkers could obtain more benefits from higher PA intensity for the lower risk of at least 1 transition from free of CMD, then to CMM, and finally to mortality.

### Interpretations, Implications, and Comparisons With Existing Literature

In line with previous studies focusing on PA amount, this study had similar findings that meeting the recommendations of PA amount could significantly decrease the risk of FCMD, CMM, and mortality, regardless of whether the traditional Cox proportional hazards regression model or multistate model was used [23-30]. This study also provided some evidence for the additional benefits of more PA (>1200 MET min/wk) for FCMD, CMM, and mortality. It is worth noting that more PA did not provide a lower risk of mortality when including CMM in the consideration of estimates from the multistate model. The aforementioned findings might be explained by the fact that the participants with CMM might have cardiovascular organic lesions and the benefits from more PA might be offset.

In line with previous studies [31-34], this study also found that higher PA intensity was significantly associated with all-cause mortality. In addition, this study added new evidence to recent knowledge that a greater proportion of VPA to MVPA (>0.75 to <1) could provide more benefits, including a decreased risk of 27% and 13% for the transitions from free of CMD to mortality and FCMD, respectively. Simultaneously, it was noted that no strong evidence was observed that a higher proportion of VPA to MVPA was associated with a lower risk of transition from free of CMD to IHD, which indicated that a suitable intensity was more effective for preventing IHD than no VPA. Another important finding in this study was that median PA intensity (the proportion of VPA to MVPA >0 to 0.75) was associated with a lower risk of transition from T2D to mortality, which suggests that stronger interventions of higher PA intensity for the participants with T2D are necessary to decrease all-cause mortality. In addition, we did not find that higher PA intensity was associated with other transitions related to CMM, which emphasizes that higher PA intensity at baseline plays an important role in the primary and secondary prevention for CMM and has a weaker role in the prognosis of CMM.

A national cohort study of >400,000 US adults reported a decreased risk of all-cause mortality associated with the synergistic effect of more PA and greater intensity [32]. This study reported similar results for the associations of an interactive effect of PA amount and intensity on transitions from free of CMD to mortality. The aforementioned associations were also significant in the transition from free of CMD to FCMD, which not only emphasized the importance of PA amount but also highlighted the importance of higher PA intensity for healthy outcomes and the development of CMM.

Inconsistent with a previous study reporting no interactions for the associations of sex with all-cause mortality [31,32], this study found that male participants could benefit more from higher PA intensity for reducing mortality from the free of CMD status. In line with the study by Wang et al [32], we also found significant interactions of current or previous smoking status and PA intensity with all-cause mortality. These differences might be caused by the different models used in this study. Instead of traditional Cox proportional hazard models used in previous studies, this study used the multistate models, which introduced a competing risk for all-cause mortality and could more validly show the transitions from free of CMD to mortality. In addition, this study provided additional evidence for identifying sensitive populations for other transitions in the incidence, progression, and prognosis of CMM, which could help develop preventive, targeted strategies for sensitive populations in the transitions.

### Strengths and Limitations

This study has several strengths. First, this study used multistate models considering the competing risk for all-cause mortality to assess the associations of PA intensity, namely the proportion of VPA to MVPA with the transitions from free of CMD to FCMD, then to CMM, and finally to mortality. Second, this study used a large-scale population–based cohort, which allowed us to explore the associations among different transitions using the prospective design. Third, long-term follow-ups of UKB in this study could provide a sufficient duration to observe the incidence, progression, and prognosis of CMM. Certain limitations should be approached with caution when interpreting the results of this study. First, the PA amount and intensity were self-reported, and more studies using portable equipment are needed to verify these associations. In addition, we only captured the baseline characteristics of PA in this study. Further studies should focus on the effects of the trajectory of PA on the transitions. In addition, although UKB had a low recruitment rate [35] and a limited age group from 37 to 73 years, a previous study proved that the associations of PA with mortality were not materially changed [45].

### Clinical Implications and Conclusions

This study suggests that higher PA intensity is an effective measure for preventing CMM and mortality in the early period of CMM development. Higher PA intensity accompanied by higher PA levels could provide the lowest risk of all-cause mortality and FCMD. Targeted populations including male participants, younger adults, people with overweight, current or previous smokers, and excessive alcohol drinkers could obtain more benefits from higher PA intensity for FCMD and all-cause mortality. Relevant interventions related to higher PA intensity should be performed.

## Appendix

Multimedia Appendix 1 Study flow, design, covariates, and additional analyses.

## Abbreviations

CMD: cardiometabolic disease
CMM: cardiometabolic multimorbidity
FCMD: first occurrence of cardiometabolic disease
HR: hazard ratio
IHD: ischemic heart disease
MET: metabolic equivalent task
MPA: moderate physical activity
MVPA: moderate to vigorous physical activity
PA: physical activity
SES: socioeconomic status
T2D: type 2 diabetes
UKB: UK Biobank
VPA: vigorous physical activity

## Data Availability

UK Biobank data can be accessed by submitting an application through the UK Biobank website.

## References

[1] (2018). Multimorbidity: a priority for global health research. The Academy of Medical Sciences.

[2] Di Angelantonio E, Kaptoge S, Wormser D, Willeit P, Butterworth AS, Bansal N, O'Keeffe LM, Gao P, Wood AM, Burgess S, Freitag DF, Pennells L, Peters SA, Hart CL, Håheim LL, Gillum RF, Nordestgaard BG, Psaty BM, Yeap BB, Knuiman MW, Nietert PJ, Kauhanen J, Salonen JT, Kuller LH, Simons LA, van der Schouw YT, Barrett-Connor E, Selmer R, Crespo CJ, Rodriguez B, Verschuren WM, Salomaa V, Svärdsudd K, van der Harst P, Björkelund C, Wilhelmsen L, Wallace RB, Brenner H, Amouyel P, Barr EL, Iso H, Onat A, Trevisan M, D'Agostino RB, Cooper C, Kavousi M, Welin L, Roussel R, Hu FB, Sato S, Davidson KW, Howard BV, Leening MJ, Leening M, Rosengren A, Dörr M, Deeg DJ, Kiechl S, Stehouwer CD, Nissinen A, Giampaoli S, Donfrancesco C, Kromhout D, Price JF, Peters A, Meade TW, Casiglia E, Lawlor DA, Gallacher J, Nagel D, Franco OH, Assmann G, Dagenais GR, Jukema JW, Sundström J, Woodward M, Brunner EJ, Khaw K-T, Wareham NJ, Whitsel EA, Njølstad I, Hedblad B, Wassertheil-Smoller S, Engström G, Rosamond WD, Selvin E, Sattar N, Thompson SG, Danesh J, Emerging Risk Factors Collaboration (2015). Association of cardiometabolic multimorbidity with mortality. JAMA.

[3] Canoy D, Tran J, Zottoli M, Ramakrishnan R, Hassaine A, Rao S, Li Y, Salimi-Khorshidi G, Norton R, Rahimi K (2021). Association between cardiometabolic disease multimorbidity and all-cause mortality in 2 million women and men registered in UK general practices. BMC Med.

[4] Cheng X, Ma T, Ouyang F, Zhang G, Bai Y (2022). Trends in the prevalence of cardiometabolic multimorbidity in the United States, 1999-2018. Int J Environ Res Public Health.

[5] Dove A, Marseglia A, Shang Y, Grande G, Vetrano DL, Laukka EJ, Fratiglioni L, Xu W (2022). Cardiometabolic multimorbidity accelerates cognitive decline and dementia progression. Alzheimers Dement.

[6] Maddaloni E, D'Onofrio L, Alessandri F, Mignogna C, Leto G, Pascarella G, Mezzaroma I, Lichtner M, Pozzilli P, Agrò FE, Rocco M, Pugliese F, Lenzi A, Holman RR, Mastroianni CM, Buzzetti R, CoViDiab Study Group (2020). Cardiometabolic multimorbidity is associated with a worse Covid-19 prognosis than individual cardiometabolic risk factors: a multicentre retrospective study (CoViDiab II). Cardiovasc Diabetol.

[7] Tai XY, Veldsman M, Lyall DM, Littlejohns TJ, Langa KM, Husain M, Ranson J, Llewellyn DJ (2022). Cardiometabolic multimorbidity, genetic risk, and dementia: a prospective cohort study. Lancet Healthy Longev.

[8] Huang Z-T, Luo Y, Han L, Wang K, Yao S-S, Su H-X, Chen S, Cao G-Y, De Fries CM, Chen Z-S, Xu H-W, Hu Y-H, Xu B (2022). Patterns of cardiometabolic multimorbidity and the risk of depressive symptoms in a longitudinal cohort of middle-aged and older Chinese. J Affect Disord.

[9] Luo H, Zhang Q, Yu K, Meng X, Kan H, Chen R (2022). Long-term exposure to ambient air pollution is a risk factor for trajectory of cardiometabolic multimorbidity: a prospective study in the UK Biobank. EBioMedicine.

[10] Lu Y, Li G, Ferrari P, Freisling H, Qiao Y, Wu L, Shao L, Ke C (2022). Associations of handgrip strength with morbidity and all-cause mortality of cardiometabolic multimorbidity. BMC Med.

[11] He L, Ma T, Li J, Luo Y, Zhang G, Cheng X, Bai Y (2022). Adherence to a healthy sleep pattern and incidence of cardiometabolic multimorbidity among hypertensive patients: a prospective study of UK Biobank. Sleep.

[12] Luo Y, He L, Ma T, Li J, Bai Y, Cheng X, Zhang G (2022). Associations between consumption of three types of beverages and risk of cardiometabolic multimorbidity in UK Biobank participants: a prospective cohort study. BMC Med.

[13] Yang L, Luo Y, He L, Yin J, Li T, Liu S, Li D, Cheng X, Bai Y (2022). Shift work and the risk of cardiometabolic multimorbidity among patients with hypertension: a prospective cohort study of UK Biobank. J Am Heart Assoc.

[14] Aune D, Norat T, Leitzmann M, Tonstad S, Vatten LJ (2015). Physical activity and the risk of type 2 diabetes: a systematic review and dose-response meta-analysis. Eur J Epidemiol.

[15] Kolb H, Martin S (2017). Environmental/lifestyle factors in the pathogenesis and prevention of type 2 diabetes. BMC Med.

[16] Smith AD, Crippa A, Woodcock J, Brage S (2016). Physical activity and incident type 2 diabetes mellitus: a systematic review and dose-response meta-analysis of prospective cohort studies. Diabetologia.

[17] Bahls M, Leitzmann MF, Karch A, Teumer A, Dörr M, Felix SB, Meisinger C, Baumeister SE, Baurecht H (2021). Physical activity, sedentary behavior and risk of coronary artery disease, myocardial infarction and ischemic stroke: a two-sample Mendelian randomization study. Clin Res Cardiol.

[18] Koolhaas CM, Dhana K, Golubic R, Schoufour JD, Hofman A, van Rooij FJ, Franco OH (2016). Physical activity types and coronary heart disease risk in middle-aged and elderly persons: the Rotterdam study. Am J Epidemiol.

[19] Winzer EB, Woitek F, Linke A (2018). Physical activity in the prevention and treatment of coronary artery disease. J Am Heart Assoc.

[20] Hung SH, Ebaid D, Kramer S, Werden E, Baxter H, Campbell BC, Brodtmann A (2021). Pre-stroke physical activity and admission stroke severity: a systematic review. Int J Stroke.

[21] Kramer SF, Hung SH, Brodtmann A (2019). The impact of physical activity before and after stroke on stroke risk and recovery: a narrative review. Curr Neurol Neurosci Rep.

[22] MacDonald CJ, Madika A-L, Gomes R, Severi G, Sibon I, Debette S, Boutron-Ruault M-C (2021). Physical activity and stroke among women - a non-linear relationship. Prev Med.

[23] Freisling H, Viallon V, Lennon H, Bagnardi V, Ricci C, Butterworth AS, Sweeting M, Muller D, Romieu I, Bazelle P, Kvaskoff M, Arveux P, Severi G, Bamia C, Kühn T, Kaaks R, Bergmann M, Boeing H, Tjønneland A, Olsen A, Overvad K, Dahm CC, Menéndez V, Agudo A, Sánchez M-J, Amiano P, Santiuste C, Gurrea AB, Tong TY, Schmidt JA, Tzoulaki I, Tsilidis KK, Ward H, Palli D, Agnoli C, Tumino R, Ricceri F, Panico S, Picavet HS, Bakker M, Monninkhof E, Nilsson P, Manjer J, Rolandsson O, Thysell E, Weiderpass E, Jenab M, Riboli E, Vineis P, Danesh J, Wareham NJ, Gunter MJ, Ferrari P (2020). Lifestyle factors and risk of multimorbidity of cancer and cardiometabolic diseases: a multinational cohort study. BMC Med.

[24] Han Y, Hu Y, Yu C, Guo Y, Pei P, Yang L, Chen Y, Du H, Sun D, Pang Y, Chen N, Clarke R, Chen J, Chen Z, Li L, Lv J, China Kadoorie Biobank Collaborative Group (2021). Lifestyle, cardiometabolic disease, and multimorbidity in a prospective Chinese study. Eur Heart J.

[25] Xie H, Li J, Zhu X, Li J, Yin J, Ma T, Luo Y, He L, Bai Y, Zhang G, Cheng X, Li C (2022). Association between healthy lifestyle and the occurrence of cardiometabolic multimorbidity in hypertensive patients: a prospective cohort study of UK Biobank. Cardiovasc Diabetol.

[26] He L, Biddle SJ, Lee JT, Duolikun N, Zhang L, Wang Z, Zhao Y (2021). The prevalence of multimorbidity and its association with physical activity and sleep duration in middle aged and elderly adults: a longitudinal analysis from China. Int J Behav Nutr Phys Act.

[27] Lee DH, Rezende LF, Joh H-K, Keum N, Ferrari G, Rey-Lopez JP, Rimm EB, Tabung FK, Giovannucci EL (2022). Long-term leisure-time physical activity intensity and all-cause and cause-specific mortality: a prospective cohort of US adults. Circulation.

[28] Samitz G, Egger M, Zwahlen M (2011). Domains of physical activity and all-cause mortality: systematic review and dose-response meta-analysis of cohort studies. Int J Epidemiol.

[29] von Rosen P, Dohrn I-M, Hagströmer M (2020). Association between physical activity and all-cause mortality: a 15-year follow-up using a compositional data analysis. Scand J Med Sci Sports.

[30] Blond K, Brinkløv CF, Ried-Larsen M, Crippa A, Grøntved A (2020). Association of high amounts of physical activity with mortality risk: a systematic review and meta-analysis. Br J Sports Med.

[31] Gebel K, Ding D, Chey T, Stamatakis E, Brown WJ, Bauman AE (2015). Effect of moderate to vigorous physical activity on all-cause mortality in middle-aged and older Australians. JAMA Intern Med.

[32] Wang Y, Nie J, Ferrari G, Rey-Lopez JP, Rezende LF (2021). Association of physical activity intensity with mortality: a national cohort study of 403 681 US adults. JAMA Intern Med.

[33] Zhao M, Veeranki SP, Li S, Steffen LM, Xi B (2019). Beneficial associations of low and large doses of leisure time physical activity with all-cause, cardiovascular disease and cancer mortality: a national cohort study of 88,140 US adults. Br J Sports Med.

[34] Rey Lopez JP, Gebel K, Chia D, Stamatakis E (2019). Associations of vigorous physical activity with all-cause, cardiovascular and cancer mortality among 64 913 adults. BMJ Open Sport Exerc Med.

[35] Sudlow C, Gallacher J, Allen N, Beral V, Burton P, Danesh J, Downey P, Elliott P, Green J, Landray M, Liu B, Matthews P, Ong G, Pell J, Silman A, Young A, Sprosen T, Peakman T, Collins R (2015). UK biobank: an open access resource for identifying the causes of a wide range of complex diseases of middle and old age. PLoS Med.

[36] Craig CL, Marshall AL, Sjöström M, Bauman AE, Booth ML, Ainsworth BE, Pratt M, Ekelund U, Yngve A, Sallis JF, Oja P (2003). International physical activity questionnaire: 12-country reliability and validity. Med Sci Sports Exerc.

[37] (2020). WHO guidelines on physical activity and sedentary behaviour. World Health Organization.

[38] Del Pozo Cruz B, Ahmadi MN, Lee I-M, Stamatakis E (2022). Prospective associations of daily step counts and intensity with cancer and cardiovascular disease incidence and mortality and all-cause mortality. JAMA Intern Med.

[39] Ding D, Van Buskirk J, Nguyen B, Stamatakis E, Elbarbary M, Veronese N, Clare PJ, Lee I-M, Ekelund U, Fontana L (2022). Physical activity, diet quality and all-cause cardiovascular disease and cancer mortality: a prospective study of 346 627 UK Biobank participants. Br J Sports Med.

[40] Textor J, van der Zander B, Gilthorpe MS, Liskiewicz M, Ellison GT (2016). Robust causal inference using directed acyclic graphs: the R package 'dagitty'. Int J Epidemiol.

[41] Tyrrell J, Jones SE, Beaumont R, Astley CM, Lovell R, Yaghootkar H, Tuke M, Ruth KS, Freathy RM, Hirschhorn JN, Wood AR, Murray A, Weedon MN, Frayling TM (2016). Height, body mass index, and socioeconomic status: mendelian randomisation study in UK Biobank. BMJ.

[42] Putter H, Fiocco M, Geskus RB (2007). Tutorial in biostatistics: competing risks and multi-state models. Stat Med.

[43] Wreede LC, Fiocco M, Putter H (2011). mstate: an R Package for the analysis of competing risks and multi-state models. J Stat Softw.

[44] Altman DG, Bland JM (2003). Interaction revisited: the difference between two estimates. BMJ.

[45] Stamatakis E, Owen KB, Shepherd L, Drayton B, Hamer M, Bauman AE (2021). Is cohort representativeness Passé? Poststratified associations of lifestyle risk factors with mortality in the UK Biobank. Epidemiology.

