# Supplementary material for: The Impact of Physical Activity Intensity on the Dynamic Progression of Cardiometabolic Multimorbidity: Prospective Cohort Study Using UK Biobank Data
Source: JMIR Public Health Surveill. 2023 Sep 25;9:e46991. doi: 10.2196/46991 (PMC10562971; doi:10.2196/46991)
Supplement: Multimedia Appendix 1 [file publichealth_v9i1e46991_app1.docx]

**Additional files**

**Figure S1:** Study flow diagram

**Figure S2:** A priori Defined Directed Acyclic Graph

**Table S1:** Data field and definition of the included covariates

**Table S2:** The associations of PA amount and intensity with FCMD, CMM and mortality

**Table S3:** The associations of PA amount with transitions from free of CMD to FCMD, CMM and mortality

**Table S4:** Subgroup analyses for the associations of PA intensity with transitions from free of CMD to specific CMD, CMM and mortality

**Table S5:** *P* values for the modifications of age, sex, BMI, PA amount, smoking and alcohol drinker status, and PA intensity with transitions from free of CMD to FCMD, CMM and mortality

**Table S6:** Sensitivity analysis for the associations of PA intensity with transitions from free of CMD to FCMD, CMM and mortality (Model A)

**Figure S1: Study flow diagram**

Participants in the present UK biobank dataset (n=502411)

Excluded participants lost to follow up or withdraw (n=1308)

Participants could be followed up (n=501103)

Excluded participants who had missing value of physical activity (n=99880)

Participants included for analyses (n=359773)

Excluded participants who had at least one cardiometabolic disease at free of CMD (n=41450)

**Figure S2:** A priori defined directed acyclic graph (DAG)

**
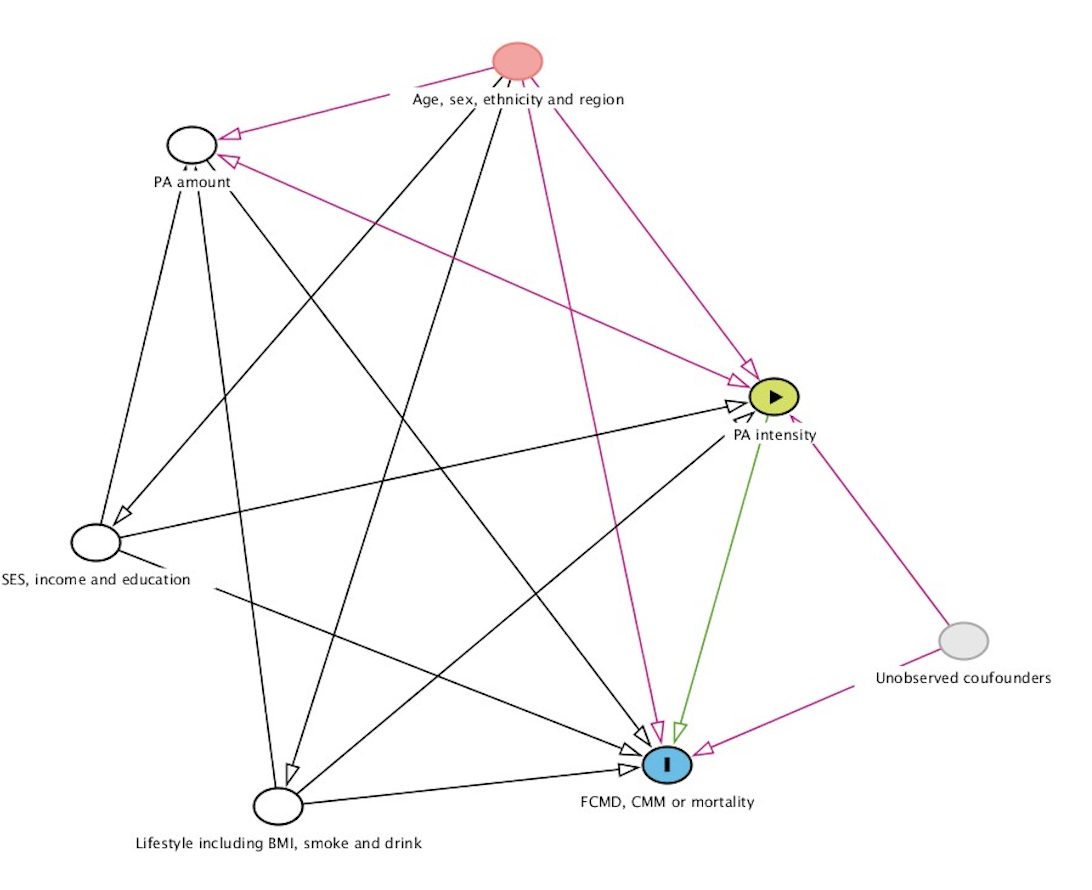
**

**
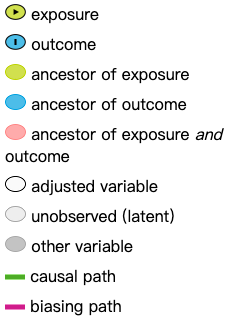
**

**Abbreviation:** BMI, body mass index; SES: socioeconomic status; PA, physical activity; FCMD, first cardiometabolic disease; CMM, cardiometabolic multimorbidity.

**Table S1:** Data field and definition of the included covariates

| **Covariates** | **Data field** | **Question and description** | **Type of variables** |
| --- | --- | --- | --- |
| Age | 21002 | Age at first recruitment | Continuous |
| Sex | 31 | Self-reported sex during the initial Assessment Centre visit | Categorical (female and male) |
| Ethnicity | 21000 | Self-reported ethnic background during the initial Assessment Centre visit | Categorical (White or others) |
| Socioeconomic status | 189 | Townsend deprivation index, which could reflect socioeconomic status, was calculated immediately prior to participant joining UK Biobank. Based on the preceding national census output areas. Each participant is assigned a score corresponding to the output area in which their postcode is located. A higher score means greater socioeconomic deprivation. | Categorical (quartiles) |
| BMI | 21001 | BMI value here is constructed from height and weight measured during the initial Assessment Centre visit. | Categorical (underweight, normal weight, overweight or obese) |
| Household income before tax per year (£) | 738 | Touchscreen question "What is the average total income before tax received by your HOUSEHOLD per year?" | Categorical (<£18000, £18000-£30999, £31000-£51999, £52000-£100000, or >£100000) |
| Study center region | 54 | The UK Biobank assessment center at which participant consented. | Categorical (England, Wales, or Scotland) |
| Education group | 6138 | Touchscreen question "Which of the following qualifications do you have? | Categorical (college or university degree; any school degree including A-level, AS-level, O-level, GCSE, CSE; vocational qualification (NVQ, HND, or HNC) or other professional qualifications; or none of the above) |
| Smoking status | 20116 | Directly use the variable 20116, which is a summary for many questions about smoking | Categorical (never, previous, or current) |
| Alcohol drinker status | 1558 | Touchscreen question "About how often do you drink alcohol?" | Categorical (daily or almost daily, above one time per week, one to three times a month, Special occasions only, never) |

**Table S2: The associations of PA amount and intensity with FCMD, CMM and mortality**

| **Variables** | **Cases/total, (n/N)** | **FCMD, HR (95%CI)** | **Cases/total, (n/N)** | **CMM, HR (95%CI)** | **Cases/total, (n/N)** | **Mortality, HR (95%CI)** |
| --- | --- | --- | --- | --- | --- | --- |
| **PA amount, (MET-mins/week)** |  |  |  |  |  |  |
| <600 | 17525/139963 | 1 | 1975/139963 | 1 | 9295/139963 | 1 |
| 600-1200 | 7269/70329 | **0.87 (0.85-0.90) †** | 741/70239 | **0.83 (0.77-0.91) †** | 3786/70239 | **0.84 (0.81-0.87) †** |
| >1200 | 16443/149481 | **0.88 (0.86-0.89) †** | 1647/149481 | **0.81 (0.76-0.86) †** | 8418/149481 | **0.81 (0.78-0.83) †** |
| **PA intensity, (the proportion of VPA to MVPA)** |  |  |  |  |  |  |
| 0 | 12306/94989 | 1 | 1379/94989 | 1 | 6811/94989 | 1 |
| >0 to 0.25 | 4867/42934 | **0.91 (0.88-0.95) †** | 471/42934 | **0.82 (0.73-0.91) †** | 2575/42934 | **0.87 (0.83-0.91) †** |
| >0.25 to 0.5 | 6808/65347 | **0.90 (0.87-0.93) †** | 666/65347 | **0.84 (0.76-0.92) †** | 3409/65347 | **0.85 (0.81-0.89) †** |
| >0.5 to 0.75 | 7340/75480 | **0.89 (0.86-0.92) †** | 653/75480 | **0.75 (0.68-0.83) †** | 3502/75480 | **0.84 (0.81-0.88) †** |
| >0.75 to <1 | 2090/24606 | **0.86 (0.82-0.90) †** | 196/24606 | **0.81 (0.69-0.95) #** | 928/24606 | **0.80 (0.74-0.86) †** |
| 1 | 899/8903 | 0.94 (0.88-1.01) | 91/8903 | 0.89 (0.72-1.10) | 424/8903 | 0.93 (0.84-1.02) |
| Ordinal scale | 34310/312259 | **0.97 (0.96-0.98) †** | 3456/312259 | **0.94 (0.92-0.97) †** | 17649/312259 | **0.96 (0.95-0.97) †** |
| *p* for trend | <0.001 | | <0.001 | | <0.001 | |

The models were adjusted for age, sex, ethnicity, socioeconomic status, BMI, household income, study center region, education group, smoking status, and alcohol drinker status. The estimates for PA intensity were further adjusted for PA amount.

† *P*<.001, # *P*<.01, **P*<.05.

Abbreviation: HR, hazard ratio; CI, confidence interval; MET, metabolic equivalent task; MVPA, moderate-to-vigorous physical activity; VPA, vigorous physical activity; FCMD, first cardiometabolic disease; CMM, cardiometabolic multimorbidity.

**Table S3: The associations of PA amount with transitions from free of CMD to FCMD, CMM and mortality.**

| **Transitions** | **Cases (n)/ total (N)** | **PA mount, MET-mins/week, (HR, 95%CI)** | | |
| --- | --- | --- | --- | --- |
|  |  | **<600** | **600-1200** | **>1200** |
| **Model A** |  |  |  |  |
| From free of CMD to FCMD | 41237/359773 | 1 | **0.86 (0.83-0.88) †** | **0.86 (0.84-0.88) †** |
| From free of CMD to mortality | 14451/359773 | 1 | **0.87 (0.83-0.91) †** | **0.84 (0.81-0.88) †** |
| From FCMD to CMM | 4363/41237 | 1 | 0.95 (0.87-1.03) | **0.90 (0.84-0.96) #** |
| From FCMD to mortality | 5991/41237 | 1 | 0.95 (0.89-1.02) | 0.95 (0.90-1.00) |
| From CMM to mortality | 1057/4363 | 1 | 1.00 (0.84-1.19) | 1.01 (0.89-1.16) |
| **Model B** |  |  |  |  |
| From free of CMD to T2D | 12059/358811 | 1 | **0.71 (0.68-0.75) †** | **0.65 (0.62-0.68) †** |
| From free of CMD to IHD | 21966/358811 | 1 | **0.93 (0.90-0.97) †** | **0.96 (0.93-0.99) #** |
| From free of CMD to stroke | 6250/358811 | 1 | **0.93 (0.86-1.00) *** | 1.01 (0.95-1.06) |
| From free of CMD to mortality | 14451/358811 | 1 | **0.87 (0.83-0.91) †** | **0.84 (0.81-0.88) †** |
| From T2D to CMM | 1202/12059 | 1 | 0.93 (0.79-1.09) | 0.94 (0.83-1.07) |
| From IHD to CMM | 1599/21966 | 1 | 0.92 (0.77-1.09) | 0.97 (0.85-1.10) |
| From stroke to CMM | 600/6250 | 1 | 0.96 (0.84-1.11) | **0.89 (0.80-0.99) *** |
| From T2D to mortality | 1123/12059 | 1 | **0.89 (0.81-0.98) *** | **0.88 (0.82-0.95) †** |
| From IHD to mortality | 3324/21966 | 1 | 0.88 (0.70-1.12) | 0.94 (0.79-1.12) |
| From stroke to mortality | 1544/6250 | 1 | 1.01 (0.88-1.17) | 0.91 (0.81-1.01) |
| From CMM to mortality | 868/3401 | 1 | 0.90 (0.74-1.09) | 1.00 (0.86-1.15) |

The models were adjusted for age, sex, ethnicity, socioeconomic status, BMI, household income, study center region, education group, smoking status, alcohol drinker status and PA amount. Model A included five transitions from free of CMD to FCMD, then to CMM, and finally to mortality (Figure 1A) and model B included eleven transitions by including specific FCMD (Figure 1B).

Abbreviation: HR, hazard ratio; CI, confidence interval; MET, metabolic equivalent task; MVPA, moderate-to-vigorous physical activity; VPA, vigorous physical activity; T2D, type 2 diabetes; IHD, ischemic heart disease; CMD, cardiometabolic disease; FCMD, first cardiometabolic disease; CMM, cardiometabolic multimorbidity.

† *P*<.001, # *P*<.01, **P*<.05.

**Table S4: Subgroup analyses for the associations of PA intensity with transitions from free of CMD to specific CMD, CMM and mortality**

| **Transitions, model B** | **Age (years old), HR (95%CI)** | | **Sex, HR (95%CI)** | | **BMI, kg/m^2^** | |
| --- | --- | --- | --- | --- | --- | --- |
|  | $\boldsymbol{\leq}$**60** | **>60** | **Male** | **Female** | **<25** | $\boldsymbol{\geq}$**25** |
| From free of CMD to T2D | **0.91 (0.89- 0.93) †** | **0.92 (0.90- 0.94) †** | **0.94 (0.92- 0.95) †** | **0.95 (0.93- 0.98) †** | **0.93 (0.89- 0.98) #** | **0.94 (0.93- 0.96) †** |
| From free of CMD to IHD | **0.97 (0.96- 0.99) †** | 0.99 (0.98- 1.01) | 0.99 (0.98- 1.01) | **0.98 (0.96- 0.99) #** | 1.00 (0.98- 1.02) | 0.99 (0.98- 1.00) |
| From free of CMD to stroke | **0.96 (0.93- 0.99) #** | **0.97 (0.95- 1.00) *** | 0.98 (0.95- 1.00) | **0.96 (0.93- 0.99) #** | 0.98 (0.95- 1.01) | **0.96 (0.94- 0.98) †** |
| From free of CMD to mortality | **0.92 (0.90- 0.94) †** | **0.94 (0.93- 0.96) †** | **0.92 (0.91- 0.94) †** | **0.96 (0.94- 0.98) †** | **0.93 (0.91- 0.95) †** | **0.94 (0.92- 0.95) †** |
| From T2D to CMM | 0.99 (0.92- 1.06) | 0.98 (0.93- 1.05) | 1.01 (0.96- 1.07) | 0.99 (0.92- 1.07) | 0.99 (0.84- 1.16) | 1.00 (0.95- 1.05) |
| From IHD to CMM | 0.96 (0.89- 1.03) | 0.96 (0.91- 1.02) | **0.94 (0.88- 1.00) *** | 1.04 (0.97- 1.12) | 1.00 (0.89- 1.14) | 0.97 (0.92- 1.02) |
| From stroke to CMM | **0.93 (0.88- 0.99) *** | 0.97 (0.92- 1.01) | 0.99 (0.95- 1.04) | 0.97 (0.91- 1.04) | 0.96 (0.88- 1.06) | 0.98 (0.94- 1.02) |
| From T2D to mortality | 0.97 (0.92- 1.01) | 0.97 (0.94- 1.01) | 0.98 (0.95- 1.01) | 1.01 (0.95- 1.06) | 0.98 (0.93- 1.03) | 0.99 (0.96- 1.02) |
| From IHD to mortality | 0.92 (0.82- 1.03) | 1.02 (0.94- 1.10) | 1.03 (0.96- 1.12) | 0.97 (0.87- 1.09) | 0.97 (0.85- 1.11) | 1.03 (0.96- 1.11) |
| From stroke to mortality | 0.94 (0.87- 1.00) | 0.97 (0.93- 1.02) | 1.00 (0.95- 1.05) | 1.00 (0.94- 1.06) | 0.96 (0.89- 1.02) | 0.99 (0.94- 1.04) |
| From CMM to mortality | **0.88 (0.79- 0.98) *** | 1.04 (0.97- 1.10) | 1.02 (0.96- 1.09) | 0.96 (0.87- 1.07) | 0.95 (0.83- 1.08) | 1.01 (0.95- 1.07) |
| **Transitions, model B** | **Smoking status (HR, 95%CI)** | | **Alcohol drinker status (HR, 95%CI)** | |  |  |
|  | **Never** | **Previous/current** | **Less** | **More** |  |  |
| From free of CMD to T2D | **0.93 (0.91- 0.95) †** | **0.94 (0.92- 0.96) †** | **0.94 (0.92- 0.95) †** | **0.95 (0.92- 0.98) #** |  |  |
| From free of CMD to IHD | 1.01 (1.00- 1.03) | **0.97 (0.96- 0.99) †** | 0.99 (0.98- 1.00) | 1.00 (0.97- 1.02) |  |  |
| From free of CMD to stroke | 1.00 (0.97- 1.02) | **0.93 (0.91- 0.96) †** | **0.97 (0.95- 0.99) *** | **0.95 (0.92- 0.99) *** |  |  |
| From free of CMD to mortality | **0.95 (0.93- 0.97) †** | **0.92 (0.91- 0.94) †** | **0.94 (0.93- 0.96) †** | **0.91 (0.89- 0.93) †** |  |  |
| From T2D to CMM | 1.00 (0.93- 1.07) | 1.00 (0.94- 1.07) | 1.00 (0.95- 1.05) | 1.02 (0.92- 1.13) |  |  |
| From IHD to CMM | 0.95 (0.88- 1.02) | 0.98 (0.93- 1.04) | 0.96 (0.91- 1.01) | 1.00 (0.91- 1.11) |  |  |
| From stroke to CMM | 0.98 (0.93- 1.04) | 0.96 (0.91- 1.01) | 0.96 (0.92- 1.00) | 1.03 (0.94- 1.12) |  |  |
| From T2D to mortality | 1.03 (0.99- 1.08) | **0.95 (0.92- 0.99) #** | 1.00 (0.97- 1.03) | 0.95 (0.90- 1.00) |  |  |
| From IHD to mortality | 1.04 (0.95- 1.14) | 0.96 (0.88- 1.05) | 1.01 (0.94- 1.09) | 0.98 (0.86- 1.11) |  |  |
| From stroke to mortality | 0.98 (0.92- 1.03) | 0.96 (0.91- 1.02) | 0.97 (0.93- 1.02) | 0.99 (0.92- 1.07) |  |  |
| From CMM to mortality | 0.98 (0.90- 1.07) | 1.01 (0.94- 1.08) | 0.97 (0.91- 1.03) | 1.07 (0.96- 1.20) |  |  |

The models were adjusted for age, sex, ethnicity, socioeconomic status, BMI, household income, study center region, education group, smoking status, alcohol drinker status and PA amount. Age, sex, BMI, smoking status and alcohol drink status were not included in the models for subgroup analyses, respectively. Model B included eleven transitions from free of CMD to specific FCMD, then to CMM, and finally to mortality (Figure 1B). PA intensity mean the proportion of VPA to MVPA, and ordinal scale of PA intensity was used in the subgroup analyses.

† *P*<.001, # *P*<.01, **P*<.05.

Abbreviation: BMI, body mass index; HR, hazard ratio; CI, confidence interval; MET, metabolic equivalent task; MVPA, moderate-to-vigorous physical activity; VPA, vigorous physical activity; T2D, type 2 diabetes; IHD, ischemic heart disease; CMD, cardiometabolic disease; FCMD, first cardiometabolic disease; CMM, cardiometabolic multimorbidity.

**Table S5: *P* values for the modifications of age, sex, BMI, PA amount, smoking and alcohol drinker status, and PA intensity with transitions from free of CMD to FCMD, CMM and Mortality**

| **Transitions** | **PA amount (600-1200 MET-mins/week)** | **PA amount (>1200 MET-mins/week)** | **Age (>60 years old)** | **Sex (Male)** | **BMI (**$\boldsymbol{\geq}$**25 kg/m^2^)** | **Smoking status (previous/current)** | **Alcohol drinker status (More)** |
| --- | --- | --- | --- | --- | --- | --- | --- |
| **Model A** |  |  |  |  |  |  |  |
| From free of CMD to FCMD | 1.000 | 0.278 | **0.006** | 1.000 | **0.030** | **0.001** | 0.278 |
| From free of CMD to mortality | 0.270 | 0.179 | 0.117 | **0.002** | 0.435 | **0.006** | **0.019** |
| From FCMD to CMM | 0.124 | 0.084 | 0.672 | 0.435 | 0.745 | 0.426 | 0.528 |
| From FCMD to mortality | 0.712 | 0.670 | 0.363 | 0.130 | 0.363 | **0.012** | 0.384 |
| From CMM to mortality | 0.887 | 0.191 | **0.004** | 0.261 | 0.147 | 0.541 | 0.102 |
| **Model B** |  |  |  |  |  |  |  |
| From free of CMD to T2D | 0.316 | 0.586 | 0.488 | 0.499 | 0.679 | 0.488 | 0.558 |
| From free of CMD to IHD | 0.219 | 0.498 | 0.063 | 0.356 | 0.379 | **<0.001** | 0.467 |
| From free of CMD to stroke | 0.271 | 0.695 | 0.615 | 0.318 | 0.275 | **<0.001** | 0.332 |
| From free of CMD to mortality | 0.270 | **0.007** | 0.117 | **0.002** | 0.435 | **0.018** | **0.019** |
| From T2D to CMM | 1.000 | 0.518 | 0.831 | 0.673 | 0.907 | 1.000 | 0.734 |
| From IHD to CMM | 0.122 | 0.079 | 1.000 | 0.039 | 0.656 | 0.510 | 0.476 |
| From stroke to CMM | 0.196 | 0.703 | 0.272 | 0.620 | 0.691 | 0.597 | 0.155 |
| From T2D to mortality | 0.802 | 0.436 | 1.000 | 0.346 | 0.738 | **0.005** | 0.097 |
| From IHD to mortality | 0.463 | 0.505 | 0.144 | 0.389 | 0.439 | 0.216 | 0.689 |
| From stroke to mortality | 0.076 | 0.437 | 0.461 | 1.000 | 0.477 | 0.615 | 0.651 |
| From CMM to mortality | 0.389 | 0.202 | **0.009** | 0.328 | 0.406 | 0.594 | 0.132 |

The models were adjusted for age, sex, ethnicity, socioeconomic status, BMI, household income, study center region, education group, smoking status, alcohol drinker status and PA amount. Model A included five transitions from free of CMD to FCMD, then to CMM, and finally to mortality (Figure 1A) and model B included eleven transitions by including specific FCMD (Figure 1B).

† *P*<.001, # *P*<.01, **P*<.05.

Abbreviation: BMI, body mass index; HR, hazard ratio; CI, confidence interval; MET, metabolic equivalent task; PA, physical activity; T2D, type 2 diabetes; IHD, ischemic heart disease; CMD, cardiometabolic disease; FCMD, first cardiometabolic disease; CMM, cardiometabolic multimorbidity.

**Table S6: Sensitivity analysis for the associations of PA intensity with transitions from free of CMD to FCMD, CMM and Mortality (Model A)**

| **Sensitivity analysis** | **Model A, (HR, 95%CI)** | | | | |
| --- | --- | --- | --- | --- | --- |
|  | **From free of CMD to FCMD** | **From free of CMD to mortality** | **From FCMD to CMM** | **From FCMD to CMM** | **From CMM to mortality** |
| Time-interval changes for the participants entering different stages at the same day |  |  |  |  |  |
| 0.5 years | **0.97 (0.97-0.98) †** | **0.94 (0.92-0.95) †** | 1.00 (0.97-1.02) | 0.99 (0.97-1.01) | 1.01 (0.96-1.06) |
| 1 year | **0.97 (0.97-0.98) †** | **0.94 (0.92-0.95) †** | 1.00 (0.97-1.02) | 0.99 (0.97-1.01) | 1.01 (0.96-1.06) |
| 2 years | **0.97 (0.97-0.98) †** | **0.94 (0.92-0.95) †** | 1.00 (0.97-1.02) | 0.99 (0.97-1.01) | 1.01 (0.97-1.06) |
| 3 years | **0.97 (0.97-0.98) †** | **0.94 (0.92-0.95) †** | 1.00 (0.97-1.02) | 0.99 (0.97-1.01) | 1.01 (0.97-1.06) |
| 5 years | **0.97 (0.97-0.98) †** | **0.94 (0.92-0.95) †** | 1.00 (0.97-1.02) | 0.99 (0.97-1.01) | 1.02 (0.97-1.07) |
| Excluding the participants who entered different stages at the same day | **0.98 (0.97-0.98) †** | **0.94 (0.92-0.95) †** | 0.99 (0.96-1.02) | 0.99 (0.97-1.01) | 1.00 (0.95-1.05) |
| Excluding FCMD occurred in the first two-year follow-ups | **0.98 (0.97-0.98) †** | **0.94 (0.93-0.95) †** | 1.00 (0.98-1.03) | 0.99 (0.97-1.01) | 1.00 (0.95-1.05) |
| Excluding the participants with cancer | **0.97 (0.97-0.98) †** | **0.94 (0.93-0.95) †** | 1.00 (0.97-1.02) | 0.99 (0.97-1.01) | 1.02 (0.97-1.07) |
| Excluding the participants with missing values at baseline for the covariates | **0.97 (0.96-0.98) †** | **0.93 (0.92-0.95) †** | 0.99 (0.97-1.02) | 0.99 (0.97-1.01) | 1.03 (0.97-1.08) |
| Further adjusting air pollution |  |  |  |  |  |
| PM_2.5_ | **0.97 (0.97-0.98) †** | **0.93 (0.92-0.95) †** | 0.99 (0.97-1.02) | 0.98 (0.96-1.01) | 1.00 (0.95-1.05) |
| PM_10_ | **0.97 (0.97-0.98) †** | **0.93 (0.92-0.95) †** | 0.99 (0.97-1.02) | 0.98 (0.96-1.01) | 1.00 (0.95-1.05) |
| NO | **0.97 (0.97-0.98) †** | **0.94 (0.92-0.95) †** | 1.00 (0.97-1.02) | 0.98 (0.96-1.00) | 1.01 (0.96-1.06) |
| NO_2_ | **0.97 (0.97-0.98) †** | **0.94 (0.92-0.95) †** | 1.00 (0.97-1.02) | 0.98 (0.96-1.00) | 1.01 (0.96-1.06 ) |

The models were adjusted for age, sex, ethnicity, socioeconomic status, BMI, household income, study center region, education group, smoking status, alcohol drinker status and PA amount. Model A included five transitions from free of CMD to FCMD, then to CMM, and finally to mortality (Figure 1A). PA intensity mean the proportion of VPA to MVPA, and ordinal scale of PA intensity was used in the sensitivity analyses.

† *P*<.001, # *P*<.01, **P*<.05.

Abbreviation: BMI, body mass index; HR, hazard ratio; CI, confidence interval; MET, metabolic equivalent task; PA, physical activity; T2D, type 2 diabetes; IHD, ischemic heart disease; CMD, cardiometabolic disease; FCMD, first cardiometabolic disease; CMM, cardiometabolic multimorbidity.
